# Chicago Medical Society

**Published:** 1881-12

**Authors:** 


### Society Reports.

### ARTICLE VI.

CHICAGO MEDICAL SOCIETY. Stated Meeting, Oct. 17, 1881.

Dr. E. Ingals, President, in the Chair.

Dr. E. L. Holmes presented an abstract of the following remarkable cases, reported by him in Knapp's Archives of Ophthalmology:

### DR. HOLMES' PAPER.

BLINDNESS—COLOR BLINDNESS DURING PREGNANCY.—Mrs. ——, thirty-one years of age, who had always enjoyed excellent health, became quite suddenly ill in the sixth month of her first pregnancy. In the course of two weeks there appeared successively symptoms of albuminurea, pericardial effusion, orthopnœa, slight but quite general œdema, and total blindness. At this stage of the disease I was requested to make an ophthalmoscopic examination.

There was an extensive neuro-retinitis of each eye. The vessels of the retina and discs, as well as the contour of the discs, were almost entirely hidden from view by exudation. The discs themselves could only be recognized as quite white flocculent clouds. At the temporal side of the right eye was a detachment of a portion of the retina. There were dilatation of the pupils and a partial paralysis of the internal rectus of the eye.

As I had often observed total atrophy of the optic nerves and total blindness in cases with a history similar to this, I could only express a doubtful prognosis as regarded the restoration of sight.

The physicians in attendance urged the induction of premature labor. I recommended the use of tinct. ferri mur., pot. iod., pot. bromide and hyd. bichlor, in suitable doses, but did not see the patient again during her illness.

Six years after this I had an opportunity of examining this patient. The left eye was almost without abnormal appearance. Vision was perfect. In the right eye, at the seat of the detachment of the retina, was a small area of pigment deposit. Central vision was nearly extinct, with great obscurity of the surrounding field.

The patient informed me that for a period the remedies which I had advised had been taken, and subsequently strychnia. During three weeks she had been totally blind, and color blind three months. Gradually she was able to distinguish white and black, then yellow, red, brown, and finally green. Not till the end of five years was there perfect faculty of perceiving tints of green.

EYE-LASH IN THE ANTERIOR CHAMBER.—A German farmer thirty-five years of age, who had lost his right eye in childhood, received a punctured wound of the left cornea from a short piece of straw thrown from a threshing-machine. Blindness of the eye was the immediate result, occsaioned probably by hæmorrhage in the anterior chamber. Little pain and inflammation followed the accident.

On examination, nine months after the injury, I observed a small opacity of the cornea at its upper and outer section. The lens was cataractous. Behind the opacity of the cornea was a rent in the iris. At the temporal portion of the anterior chamber was an eye-lash, which had evidently been carried through the cornea by the piece of straw. This was easily removed by means of a small blunt hook, through a minute incision in the cornea. A subsequent removal of the cataract restored excellent vision.

Dr. Holmes also reported the following case of anchylo-blephoran:

Last summer he was called to see a child four years old, in whom both eyelids were so covered with sores that it seemed impossible as well as inexpedient to separate them. The child had been vaccinated in the latter part of the winter; from that time herpetic sores had broken out all over the skin; but to what extent the vaccination was accountable for the disease, Dr. H. could not determine. The patient presented all the indications of scrofula, and was put on appropriate treatment. He saw the case a few days ago, and noticed the following conditions: The skin had been restored, but the eyelids had not been separated. These he parted by inserting the points of scissors into a small incision made with a small bistoury between what had been the edges of the lids. The cornea of one eye was opaque, in the other quite transparent. There was some photophobia. Dr. Holmes remarked that sewing the eyelids together, and in due time separating them again, are recommended in xerophthalmia.

Dr. Hotz had met with cases of neuro-retinitis and blindness complicating pregnancy. Sight was restored in many cases, and as to the prognosis, he believed that it was much more favorable whenever blindness was dependent on the albuminous retinitis of pregnancy than on that of Bright's disease; and in the latter case it was always a precursor of death.

Dr. R. Tilley reported a case of strabismus depending on inflammation of the middle ear, which ceased after the discharge from the ear had stopped.

Dr. Holmes gave his assent to Dr. Hotz' remarks, but he added that rather a large proportion of cases of blindness from pregnancy had come under his observation which did not recover.

Dr. Hotz reported some cases of wounds of the cornea by straws from threshing-machines. It was a most frequent cause of injury among farmers. The pieces of straw should always be extracted, even when the cornea had to be cut into for that purpose, and the use of chloroform was advisable when required.

Dr. Ingals asked of the ophthalmologists present, what was the cause of asthenopia after labor? He had always supposed that it depended on straining of the ophthalmic nerves in the straining of labor, and on the congestion then liable to be present.

Dr. Holmes did not think that it was owing to congestion as much as to temporary weakness of the muscles of accommodation following exhaustion of the system at large. But this explanation was not satisfactory to the enquirer.

Dr. Tilley related an interesting case in a girl sixteen years of age, who had not opened her eyes for two months when he first saw her. She had a tumor of the mammary gland, which was removed, and found to be a cystic sarcoma. Just as she recovered from under the influence of the anæsthetic, she opened her eyes. After four weeks, the same trouble returned, and the patient was again anæsthetized, with the same result. After two months there was marked internal strabismus, which was overcome by straining the eye in the opposite direction repeatedly.

Dr. Emma F. Gaston, six weeks ago, had seen a lady in apparent good health, who was suddenly attacked by irritation of the eyes while paring peaches. The inflammation had subsided, under proper treatment, in three days. Was there something irritating or poisonous about the peaches ?

